# Polyurethane Composites Recycling with Styrene–Acrylonitrile and Calcium Carbonate Recovery

**DOI:** 10.3390/ma17122844

**Published:** 2024-06-11

**Authors:** Jesús del Amo, Subramaniam Iswar, Thomas Vanbergen, Ana Maria Borreguero, Simon Dirk E. De Vos, Isabel Verlent, Jan Willems, Juan Francisco Rodriguez Romero

**Affiliations:** 1 Chemical Engineering Department, University of Castilla-La Mancha, Institute of Chemical and Environmental Technology, ITQUIMA, Avda. Camilo José Cela s/n, 13004 Ciudad Real, Spain; jesus.delamoleon@geacam.com (J.d.A.); anamaria.borreguero@uclm.es (A.M.B.); 2 Recticel Engineered Foams Belgium BV, Damstraat 2, 9230 Wetteren, Belgium; iswar.subramaniam@recticel.com (S.I.); verlent.isabel@recticel.com (I.V.); willems.jan@recticel.com (J.W.); 3 Centre for Membrane Separations, Adsorption, Catalysis and Spectroscopy for Sustainable Solutions (cMACS), KU Leuven, Celestijnenlaan 200F, P.O. Box 2454, 3001 Leuven, Belgium; thomas.vanbergen@kuleuven.be (T.V.); dirk.devos@kuleuven.be (S.D.E.D.V.)

**Keywords:** glycolysis, flexible polyurethane foams, polyurethane composites, styrene–acrylonitrile, calcium carbonate, physical and mechanical properties

## Abstract

The glycolysis process of flexible polyurethane foams containing styrene–acrylonitrile and calcium carbonate as fillers was explored in detail. The use of DABCO as a catalyst allowed us to reduce the catalyst concentration and the polyurethane-to-glycol mass ratio to 0.1% and 1:1, respectively. The glycolysis process allowed us to obtain a high-purity polyol (99%), which can totally replace raw polyols in the synthesis of new flexible polyurethane foams, maintaining the standard mechanical properties of the original one and modifying the ratio of isocyanates employed to correct the closed cell structure caused by the impurities present in the recovered polyol. This isocyanate mixture was also optimized, resulting in a ratio of 30 and 70% of the isocyanates TDI80 and TDI65, respectively. Additionally, the fillers incorporated in the glycolyzed foams were recovered. Both recovered fillers, styrene–acrylonitrile and calcium carbonate, were fully characterized, showing a quality very similar to that of commercial compounds. Finally, the replacement of commercial fillers by the recovered ones in the synthesis of new polyurethane foams was studied, demonstrating the feasibility of using them in the synthesis of new foams without significantly altering their properties.

## 1. Introduction

Polyurethanes (PUs) are usually thermoset polymers with a production of 27 million tons per year in 2021, being the polymer group with the seventh highest production and consumption in the world [1,2,3]. Polyurethane synthesis occurs by mixing two main compounds, polyol and isocyanate, giving rise to a nucleophilic addition reaction between a multifunctional alcohol or polyol and di/tri-isocyanate, obtaining a reticular structure called polyurethane [4,5,6].

Polyurethanes can be classified as a function of their applications in two main groups, foams and CASEs (coatings, adhesives, sealants and elastomers) [4]. Moreover, the foams can be subdivided into flexible and rigid foams, where flexible foams are employed in applications such as comfort, packaging and car seats, and rigid foams are used in refrigeration and construction materials [4]. Among the polyurethane types, flexible and rigid foams have the highest production volumes due to their wider applications and success, close to 70% of the production of these materials. Due to the great commercial success and multitude of applications, a large amount of waste is generated, which generally ends up in landfills, generating a heavy environmental impact. Therefore, the development of a polyurethane recycling process is essential to make their consumption and application fulfil the concepts of a circular economy.

For the synthesis of polyurethane foams, two main reactants, polyol and isocyanate, are employed, and additives like catalysts, surfactants, antioxidants and fillers are incorporated in order to obtain products with suitable properties for the market demand [7,8,9,10]. In the case of flexible polyurethane foams, fillers, such as calcium carbonate, are often used to obtain better firmness, resistance to impact, less flammability, less fragility and even a smoother and durable surface [11,12]. The use of inorganic fillers, such as calcium carbonate, also implies an increase in the foam density, which leads to an increase in hardness, but properties such as traction and elongation are worsened [13]. On the other hand, it is very common to use polyols with chemically grafted styrene–acrylonitrile (SAN) microparticles, which allows one to create polyurethane foams with better hardness without deteriorating the rest of the mechanical properties [13,14].

Nowadays, it is very common to find materials improved with fillers or composites; so, it is also crucial to design a recycling process for these materials. This research is focused on demonstrating the feasibility of recycling flexible polyurethane foams containing calcium carbonate and SAN, allowing the recovery of both the polyol and the fillers from those end-of-life foams.

Recycling processes can be divided into physical or chemical processes, the latter being the most interesting since they allow the recovery of the polyol and/or other products derived from isocyanate, which could be used for the partial or total replacement of fresh ones [15,16]. Thus, the chemical processes constitute the best alternative for recycling these polyurethane foams because they allow one to obtain the initial raw materials, reducing the depletion of scarce sources and avoiding more polluting processes.

**Scheme 1:**
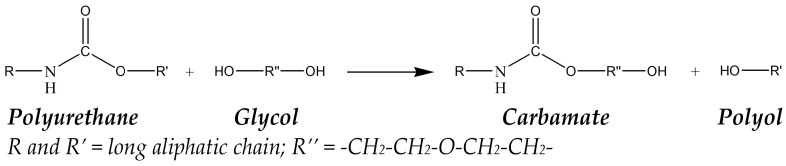
Polyurethane glycolysis reaction.

There are different chemical processes, such as hydrolysis, aminolysis, phosphorolysis, glycolysis, etc. The most advisable one is glycolysis, as this process allows one to obtain the recovered products with highest purity using mild reaction conditions [17,18,19]. Moreover, the glycolysis process is the best developed technique. It consists of a reaction between urethane and glycol to form polyol, carbamate, secondary amine and carbon dioxide [17,18,20]. The glycolysis reaction of polyurethane foams with a glycol is presented below (Scheme 1).

Furthermore, when a large excess of glycol is used for glycolysis, the reaction system splits into two immiscible phases, the apolar polyol-rich phase and the glycolysis agent-rich phase. In previous studies, diethylene glycol or crude glycerol and stannous octoate with a mass ratio to polyurethane of 1.5 to 1 were used in order to obtain a product with a split phase. The reaction conditions employed in this research have been previously optimized by our research group [21,22,23,24] and by the European consortium PUReSmart. In the glycolysis of polyurethane foams containing calcium carbonate and SAN, a product with four different phases (upper, intermediate, bottom and solid phase) was obtained. The upper phase, consisting mainly of the recovered polyol, had a higher purity than the liquid obtained by means of processes without a split phase. The intermediate phase contained a high concentration in SAN, which after an exhaustive purification process allows one to obtain the recovered copolymer with high purity. The bottom phase showed high concentration in the glycolysis agent and the reaction by-products. Finally, the solid phase was composed mainly by the inorganic filler, specifically the recovered calcium carbonate, as was the case with other inorganic fillers in the glycolysis of other polyurethane foams previously discussed [25]. To the best of our knowledge there are no previous works describing the recovery of the components of a PU foam residue containing SAN microparticles and calcium carbonate as fillers. Moreover, the recovered products have been reused in the synthesis of new flexible polyurethane foams, leading to new materials with suitable properties for commercial applications.

## 2. Materials and Methods

### 2.1. Materials

Foam samples formulated with polymeric polyether polyols containing styrene–acrylonitrile (SAN) particles and calcium carbonate (CaCO_3_) as the filler were supplied by Recticel (Wetteren, Belgium) and shredded in small pieces of about 1 cm^3^ to facilitate their feeding to the reactor. The glycolysis medium was composed of a glycol and a catalyst, diethylene glycol (purity 99.8%, supplied by Campi y Jové S.A., Barcelona, Spain) and 1,4-diazabicyclo octane [2.2.2] (DABCO of purity 99%, supplied by Sigma-Aldrich, Madrid, Spain).

Moreover, methanol (purity 99.8%, supplied by Labkem, Barcelona, Spain), acetone (purity 99.6%, supplied by Labkem, Barcelona, Spain), cyclohexane (purity 99.9%, supplied by Labkem, Barcelona, Spain) and milli-Q water (resistivity of 18.2 MΩ·cm) were employed in the extraction and purification processes.

Finally, polyurethane foams were synthesized employing pure or/and recovered polyols, pure or recovered fillers such as SAN and CaCO_3_, toluene diisocyanate (TDI), water as the blowing agent, a standard catalyst and a standard surfactant.

### 2.2. Glycolysis Reaction Process

Glycolysis reactions of flexible polyurethane foams containing SAN and CaCO_3_ as fillers were carried out on a laboratory scale with a two-liter volume reactor. The glycolysis unit presented a jacketed reactor heated with silicone oil employing a thermostatic bath. Moreover, the reactor had at the top a condenser, a nitrogen intake to ensure inert atmosphere and avoid oxidation, an additional mouth to add the glycol and the catalyst and a stirring head to drive a six-blade Rushton-type agitator. It was also provided with a bottom valve, used for taking samples during the reaction and for the discharge of the reaction product. The installation was placed in a fume extraction hood. Once the reaction temperature of 200 °C was reached, the polyurethane foam wastes were fed by an automatic feeder in a time of one hour. The stirring speed was of 300 rpm to ensure complete homogenization. The reaction conditions employed were a mass ratio of PU to glycolysis agent of 1:1, a catalyst concentration of 0.1 wt%, a feeding time of 1 h and a reaction time of 3 h. After the reaction time, the glycolyzed product was recovered and left to decant in a funnel to separate the different phases. A summary of the main reaction conditions and the reaction recipe employed in the glycolysis process are presented in Table 1.

### 2.3. Extraction and Purification Processes

The liquid–liquid extraction installation consisted of a 2-liter flask, thermostated with silicone oil coming from a recirculation thermostat bath. Moreover, the installation consisted of a reflux condenser, a temperature control system and a digital stirring head Heidolph RZR 2041 (Mervilab, Madrid, Spain), with an agitation range of 40–2000 rpm, which powers a 6-pallet Rushton-type agitator.

The purification of the upper phase obtained after glycolysis was performed by means of two extractions with cyclohexane and two washes with neutral water. The conditions of the extractions and washes were a temperature of 60 °C, an agitation of 300 rpm, a time of a stirring time of 30 min, a settling time of 15 min for extractions, 60 min for washes and a ratio of upper phase to cyclohexane for extractions of 1 to 1.5, and for washes, of 1 to 1. Lastly, the cyclohexane and the water were removed completely in a rotary evaporator obtaining the recovered polyol, employing a temperature up to 180 °C and a vacuum of 20 mbar.

The recovered styrene–acrylonitrile copolymer intermediate phase was washed with two aliquots of methanol at room temperature with a mass ratio of the intermediate phase to methanol of 1 to 1. The methanol was removed by filtration under vacuum and the solid obtained was dried in an oven at 70 °C to ensure the complete elimination of the solvent.

Finally, the purification of the solid phase was carried out by two washes with acetone, allowing us to obtain the recovered calcium carbonate. The mass ratio of the solid phase to acetone was 1 to 1 and the extraction was carried out at room temperature. The acetone was removed analogously to the methanol in the purification of the intermediate phase.

### 2.4. Polyurethane Synthesis

Flexible foaming tests were performed in standard formulations for flexible foams, based on a polyether polyol (viz. poly(propylene oxide)-block-poly(ethylene oxide) with functionality 3) combined with recycled polyether polyols and/or recovered fillers. Toluene diisocyanate (TDI) was used as the isocyanate, specifically TDI80 and/or TDI65, water was employed as the blowing agent and a standard catalyst (amine and metal catalysts) and a standard surfactant were used to obtain flexible PU foams. The reaction time or blow-off time to obtain these foam samples was recorded. Table 2 shows the recipe of the synthesized foams in the absence of fillers, expressed in weight in parts per polyol (p/p w).

### 2.5. Characterization Techniques

#### 2.5.1. Molecular Weight and Product Composition Determination by Gel Permeation Chromatography (GPC)

The molecular weight, the molecular weights distribution (MWD) and the purity of the recovered products were determined by GPC. The GPC equipment was a Viscotek GPCmax VE-2001 TDA 302 Detectors chromatograph (IESMAT, Alcobendas, Madrid, Spain) with two peristaltic pumps, an automatic injection system, an electric oven, two columns (Water Styragel Column HR2 (pore size 500Å, molecular weight 0 to 100 g/mol) and HR0.5 (pore size 50Å, molecular weight 500 to 20,000 g/mol)) (from Waters Cromatografía, S.A., Cerdanyola del Vallès Barcelona, Spain) and triple detection, consisting of an LALS (Low-angle light scattering) detector, a RALS (right-angle light scattering) detector and a viscosity detector. OmniSEC 4.5.6 is the program available in the GPC equipment for recording and analyzing the results. The conditions were a temperature of 40 °C, a flow rate of 1ml/min, a sample concentration of 10 mg/mL and an injection volume of 100 µL. Poly(ethylene glycol) standards (from Waters Cromatografía, S.A., Cerdanyola del Vallès Barcelona, Spain) were used for MWD calibration.

#### 2.5.2. Fourier-Transform Infrared Spectroscopy (FTIR)

Functional groups of the recovered products were determined by infrared analyses, employing a Varian 640-IR FT-IR (Mervilab, Madrid, Spain) spectrophotometer in the range of 4000 to 400 cm^−1^, 8.0 cm^−1^ resolution and 16 scans, with a program called Varian Resolution Pro Software, version 5.0.

#### 2.5.3. Measurement of Hydroxyl Index (iOH)

The hydroxyl number of the recovered polyol was determined by a standard titration method (AOCS Official Method Cd 13-60) [26].

#### 2.5.4. Viscosity

Rotational rheometry is a technique used to study the shear rheology of fluids, in this case, of the recovered polyol. This technique studies the behavior of the fluid when it is subjected to a shear stress, allowing the detection of changes in the structure of the materials and determining their viscosity. The equipment used was a BOHLIN GEMINITM 200 rheometer from Malvern Instruments (IESMAT, Alcobendas, Madrid, Spain), consisting of a cone/plate system, in this case, a 4/60 cone. Samples are placed on a plate with control of the temperature, while the cone is driven by an ultra-low inertia motor coupled to an ultra-high precision position in order to minimize measurement disturbance. The measurements were carried out by controlling the shear rate between 0 and 680 s^−1^.

#### 2.5.5. Water Content

Water contents for the recovered polyols were determined by the Karl Fischer method using an automatic titrator Titrino KF, according to the standard ASTM D-4672-12 [27]. The equipment works employing methanol in which the sample is solubilized. Firstly, the device prepares the methanol to neutralize the water contained in the sample by means of the Karl Fischer Hydranal Composite 5 reactive agent Riedel de Haën (Mervilab, Madrid, Spain). Then, a known quantity of the sample is added, and the water content is automatically determined by means of titrating with the Karl Fischer reactive agent, obtaining the final result as water percentage (%).

#### 2.5.6. X-ray Diffraction (XRD)

The crystal structure of the recovered calcium carbonate was analyzed by using an X-ray diffractometer (Philips, Amsterdam, The Netherlands) model X, Pert MPD. The equipment had a radiation Cu-Kα, automatic divergence slit, graphite monochromator and xenon gas sealed detector.

#### 2.5.7. Foams Characterization

The apparent density was measured according to the ISO 845 standard [28]. The air resistance was measured according to the ISO 9237 standard [29]. The air resistance measures the velocity of an air flow passing perpendicularly through the sample (minimally 150 × 150 mm^2^) with a thickness of 10mm and a pressure drop of 200 Pa. The compression load deflection (CLD) hardness 40% was measured according to the ISO 3386/1 standard [30]. The CLD hardness 40% is defined as the force required to compress a 100 × 100 × 50 mm piece of foam with a 200 mm diameter compression plate. The foam is compressed three times to 70%, and after the third compression, a reading is taken at 40%. The hysteresis (energy) loss is defined as the difference between the loading energy and the unloading energy expressed as a percentage of the loading energy. The compression set 50% was measured according to the ISO 1856A standard [31]. The compression set 50% measures the permanent changes in the thickness of a sample after a compression of 50% at 70 °C during 22h; it is expressed in percent compared to the initial thickness.

## 3. Results

### 3.1. Glycolysis Process of Flexible PU Foams Containing CaCO_3_ and SAN as Fillers

The reaction conditions employed in this glycolysis process were those presented in Table 1, together with the reaction recipe.

**Figure 1:**
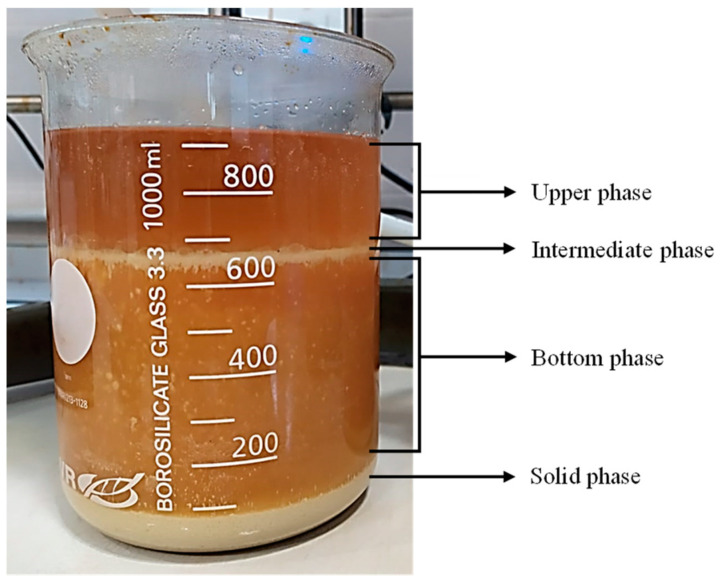
Glycolysis mixture obtained after reaction.

Once poured into a laboratory glass and cooled down, the glycolysis mixture presented the aspect shown in Figure 1.

**Figure 2:**
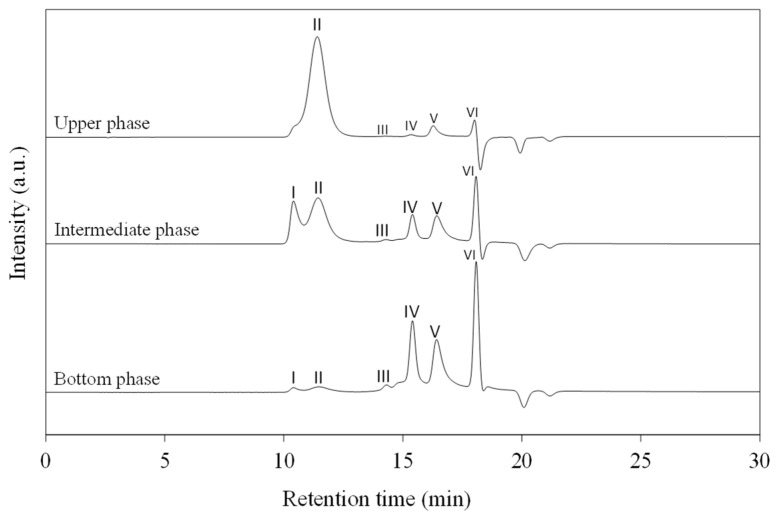
GPC chromatograms of the upper, intermediate and bottom phases at the end of reaction. Peak I = SAN; Peak II = flexible polyether polyol; Peaks III, IV and V = reaction by-products; Peak VI = DEG.

A visual inspection of the glycolysis reaction product allows us to appreciate four phases, an upper phase (UP), an intermediate phase (IP), a bottom phase (BP) and a solid phase (SP), which are expected to correspond mainly to the recovered polyol, the SAN copolymer, the excess of diethylene glycol and isocyanate-derived by-products and the settled CaCO_3_, respectively. A GPC analysis of the different phases was carried out to associate each phase with its expected composition (Figure 2), except for the case of the solid phase or calcium carbonate since it is an inorganic compound and is not soluble.

The GPC results allowed us to determine the concentration and molecular weights corresponding to the compounds related to each peak (Table 3), confirming the nature of each peak. These results confirmed the successful glycolysis of the PU foams, leading to an upper phase composed mainly by the recovered polyol (Peak II), together with small impurities of the reaction by-products (Peaks III–V) and diethylene glycol (Peak VI). The product concentrations were determined from the chromatogram areas (Table 3) [32]. The high polyol purity, close to 90 wt%, is due to the difference in polarity between the polyol and DEG and the rest of isocyanate-derived products. For the same reason, the GPC result of the bottom phase shows the low solubilization of SAN and polyol (Peaks I and II, respectively), being mainly formed as reaction by-products from isocyanate, such as carbamates and secondary amines (Peaks III–V) and diethylene glycol (Peak VI). This bottom phase could be used in the synthesis of rigid polyurethane foams as a partial replacement for the raw rigid polyol as demonstrated in previous research [23]. Finally, the intermediate phase showed a high concentration in SAN. However, it also presented high contamination from the other phases, which would entail a purification process to obtain the recovered SAN with a higher purity to be reused.

**Figure 3:**
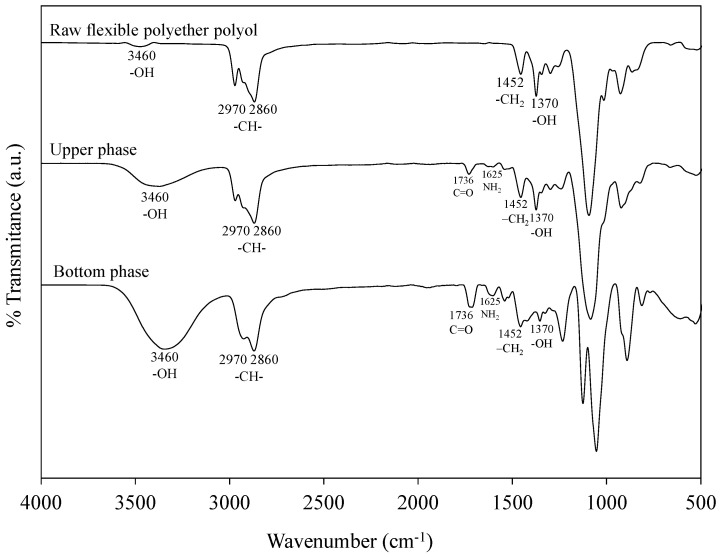
FTIR spectra of the commercial polyol and the UP and BP obtained after reaction.

Figure 3 shows the FTIR spectra of a trifunctional polyether polyol with a molecular weight of 3500 g/mol and of the UP and BP obtained after reaction.

It can be seen in Figure 3 that the spectra of the commercial polyol show the same signals as the upper phase, corresponding to the hydroxyl groups (-OH) at 3460 cm^−1^, aliphatic carbons of the chain polyol (-CH) at 2860–2970 cm^−1^, methylene groups (-CH_2_) at 1452 cm^−1^, and the alcohol group of polyol at 1370 cm^−1^ [33]. However, in the case of the upper phase, there are also two small signals from the by-products of the glycolysis, corresponding to the carbonyl group (C=O) of the carbamates at 1736 cm^−1^ and the amine groups (NH_2_) at 1625 cm^−1^ [33]. The low intensity of these signals indicates that the concentration of by-products is quite low in the upper phase. Another remarkable result is that the signal intensity of the hydroxyl groups in the upper phase is higher than in the commercial polyol, indicating a higher concentration of functional hydroxyl groups in this phase due to the presence of diethylene glycol. On the other hand, the FTIR spectra of the bottom phase show the same peak structure as the upper phase, but the signal intensities of the alcohol, amine and anhydride groups are larger than in the upper phase, agreeing with the fact that this phase is composed mainly of reaction by-products and diethylene glycol.

On the other hand, from the mass balance of glycolysis and purification processes, it is possible to estimate that the recovery yields of polyol, SAN particles and CaCO_3_ are 94, 63 and 74%, respectively.

Therefore, it has been demonstrated that it is possible to carry out the glycolysis process of flexible polyurethane foams containing SAN microparticles and calcium carbonate as the filler, obtaining a split-phase product with properties similar to those of other recovered polyols, reducing the ratio of glycol to PU from 1.5 to 1 and the catalyst concentration from 1.3% to 0.1%. These reaction conditions present better technical and economic perspectives.

### 3.2. Purification and Characterization of Recovered Polyol

**Figure 4:**
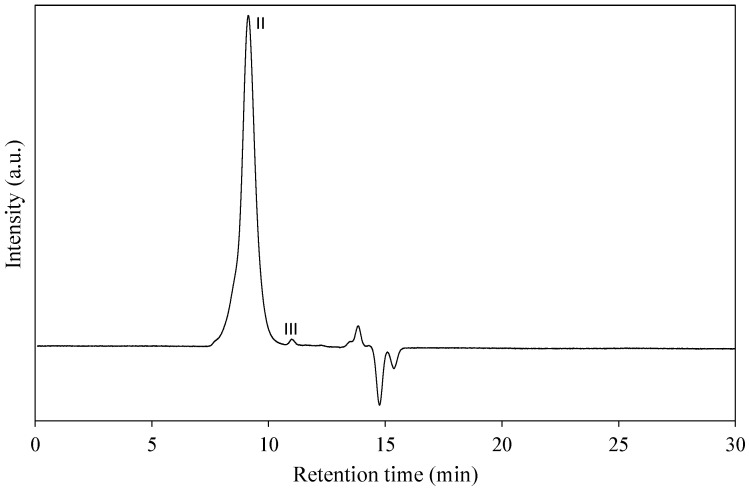
GPC chromatogram of the purified recovered polyol. Peak II = flexible polyether polyol; Peak III = reaction by-product.

The upper phase was purified as described in Section 2.3, and the recovered polyol obtained was characterized by GPC (Figure 4).

Once purified, the polyol obtained contains a lower concentration of reaction by-products than the upper phase obtained after the glycolysis process, and the glycolysis agent has been totally removed (Peak VI).

The composition of the purified polyol, based on the GPC results, is presented in Table 4, along with other physicochemical parameters such as the hydroxyl number, the water content and viscosity.

The glycolysis process has been demonstrated to be very robust. The recovered polyol obtained from PU foam residues showed a purity higher than 99%. Therefore, it can be used for the synthesis of new polyurethane foams.

### 3.3. Synthesis and Characterization of Flexible Polyurethane Foams Using the Recovered Polyol

Initially, two different foams were synthesized, one with virgin polyol (reference) and the other with the recovered one, without altering the rest of the values of the recipe presented in Section 2.4.

Both foams showed adequate growth, as well as an acceptable density value of 25 kg/m^3^, in the case of the one that used virgin polyol, and 22.9 kg/m^3^, when using recovered polyol. However, the foam obtained with the recovered polyol presented a closed cell structure, which led to foam with a high resistance to air (85 cm H_2_O), while the foam synthesized with virgin polyol presented a value of 6.2 cm H_2_O and therefore an open-cell structure [34]. The closed-cell structure of the foam obtained using the recovered polyol was due to the presence of reaction by-products in this recovered product, which presented a purity of 99% by GPC, that promote higher reactivity. This is also reflected in properties like amine value, hydroxyl number and viscosity, which were not identical to those of a fossil fuel-based conventional polyol. However, the foams that used up to 50% recovered polyol did not present such a closed-cell structure and had air resistance values similar to the reference [35].

To avoid the closed-cell structure with the increase in the recovered polyol content in the foam recipe, the possibility of partially or fully replacing the isocyanate TDI80 by TDI65 was studied, with the aim of synthesizing foams with an open-cell structure and therefore a lower air resistance value.

Table 5 shows the air resistance values of each foam synthesized with 100% of the recovered polyol and different isocyanate mixtures of TDI80 and TDI65.

From these results, it can be concluded that the optimal ratio of the isocyanate mixtures are 70% TDI65 and 30% TDI80 since the resulting foam approximates the value of air resistance of the reference foam. In the case of exclusively using the TDI65 isocyanate, the air resistance value was lower, but the foam did not grow properly. Previous investigations proved that the use of isocyanate TDI65 led to the formation of urea precipitates greater than those caused by TDI80. The formation of these urea aggregates is related to the opening of the cells of flexible polyurethane foams. For this reason, in the previous research, the foams synthesized with TDI65 showed all their cells of the open structure, as was not in the case with the foams obtained using TDI80 [36]. Furthermore, the 2,6-TDI isomer has lower reactivity than 2,4-TDI. Therefore, using a greater amount of TDI65, the amount of the 2,6-TDI isomer is increased, thus decreasing reactivity and favoring the slower growth of the foam and therefore an open-cell structure of the foams [37]. These reasons explain why the use of TDI65 leads to obtaining flexible polyurethane foams with an open-cellular structure and therefore with lower air resistance.

Finally, polyurethane foams were synthesized with different proportions of virgin and recovered polyol and using the identified optimum isocyanate ratio (TDI65/TDI80 of 70/30) to study their physical and mechanical properties and compare them to those of the reference foam (Table 6).

When the recovered polyol content increased from 50% to 100%, a significant amount of isocyanate TDI65 was required to open the foam (Table 5). This resulted in high hardness and high wet compression set (WCS) values (Table 6), due to which the final foam properties were out of specification, which was finally linked to the impurities (amines) present in the recovered polyol. For 50, 60 and 70% of the recycled polyol, the partial substitution of TDI80 by TDI65 allowed us to obtain foams within the specifications. It should be noted that amines or impurities present in the recovered polyol could catalyze gas formation or chain extension reactions, causing faster foam growth [38,39]. Therefore, the addition of the TDI65 isocyanate with a greater proportion of the 2,6-TDI isomer, less reactive than 2,4-TDI [36,37], corroborates the obtaining of foams with adequate properties, since the increase in reactivity with the impurities present in the recovered polyol is counteracted.

### 3.4. Purification and Characterization of the Recovered Styrene–Acrylonitrile Copolymer

A separation and purification method of the intermediate phase was developed with the aim of recovering the SAN copolymer.

**Figure 5:**
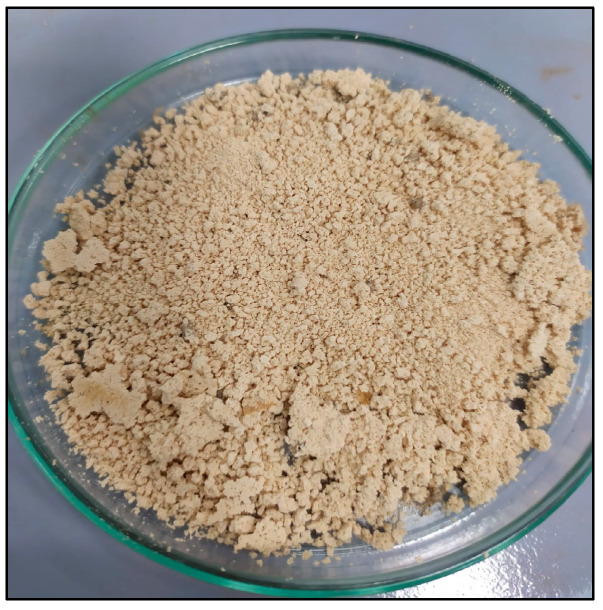
Visual appearance of recovered SAN.

The intermediate phase was washed twice with methanol aliquots at room temperature; after that, it was filtered under vacuum and dried in an oven at 70 °C, obtaining the recovered SAN depicted in Figure 5.

**Figure 6:**
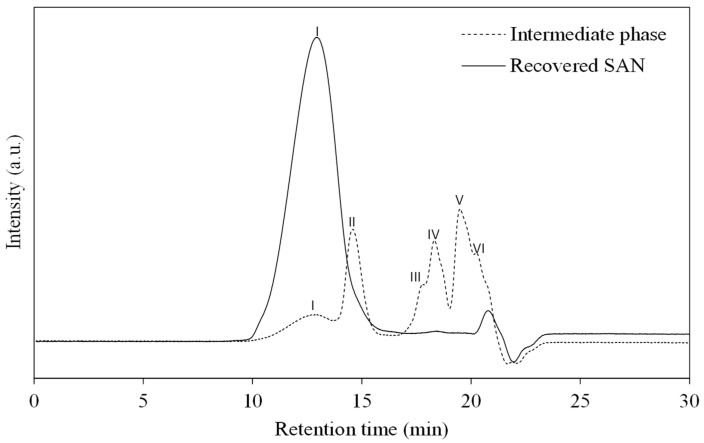
GPC chromatograms of recovered SAN and of intermediate phase at the end of reaction. Peak I = SAN; Peak II = flexible polyether polyol; Peaks III, IV and V = reaction by-products; Peak VI = DEG.

Figure 6 shows the GPCs of the purified SAN in comparison with that of the intermediate phase.

According to the GPC results, after the purification process, there was just pure SAN, without impurities, since just one peak, corresponding to the SAN retention time, appeared in the chromatogram.

**Figure 7:**
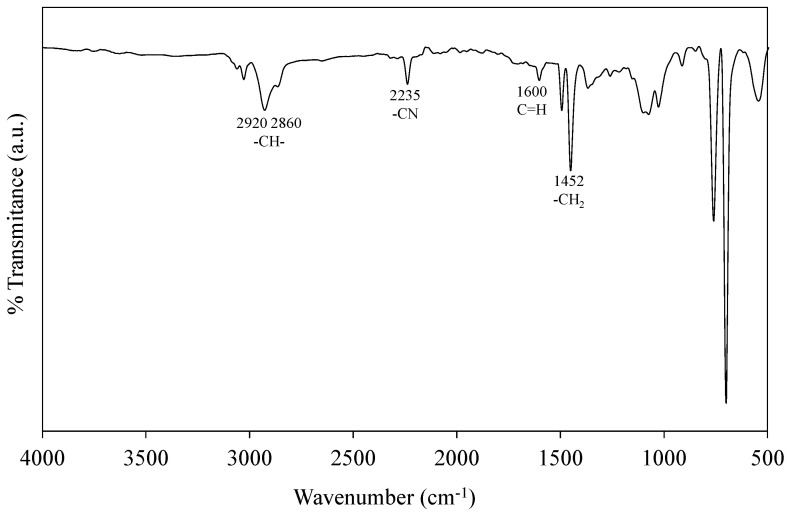
FTIR analysis of recovered SAN.

The recovered SAN characterization was completed by means of infrared analysis (Figure 7).

This spectrum was compared with data from a bibliography presenting complete coincidence [13,40], with signals corresponding to the aromatic ring of the phenyl group (C=C) at 1600 cm^−1^, the nitrile group originating from the acrylonitrile (-CN) at 2235 cm^−1^, the aliphatic carbons of the polymer chain (-CH, CH_2_) at 2920–2860 cm^−1^ and the methylene groups (-CH_2_) at 1452 cm^−1^ [33]. Therefore, the infrared analysis corroborated the GPC result in terms of the high quality of recovered SAN. This enables its use in the synthesis of new polyurethane foams as fillers or even in other applications.

### 3.5. Purification and Characterization of Recovered Calcium Carbonate

**Figure 8:**
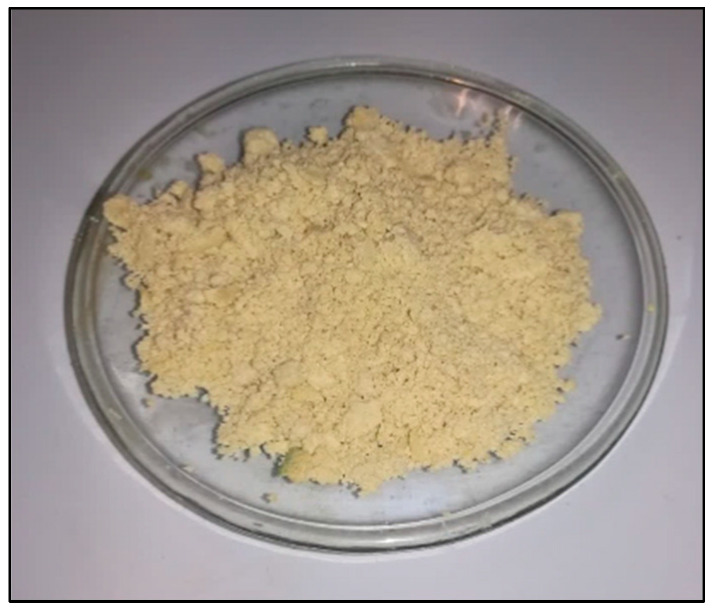
Appearance of recovered calcium carbonate.

The calcium carbonate precipitate was also purified in order to demonstrate its reusability. The purification procedure consisted in two washes with acetone, filtering under vacuum and drying in the oven at 70 °C, obtaining the recovered filler as shown in Figure 8.

**Figure 9:**
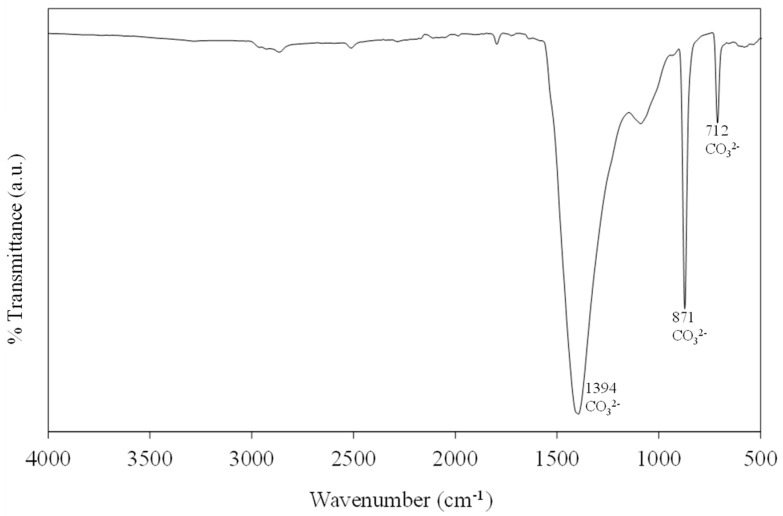
Infrared analysis of recovered calcium carbonate.

Figure 9 shows the characterization of the recovered calcium carbonate by FTIR.

It can be concluded that the purified solid product was pure CaCO_3_ since the spectrum presented only the signals corresponding with the carbonate groups (CO_3_^2−^) at 1394, 871 and 712 cm^−1^ [33,41].

**Figure 10:**
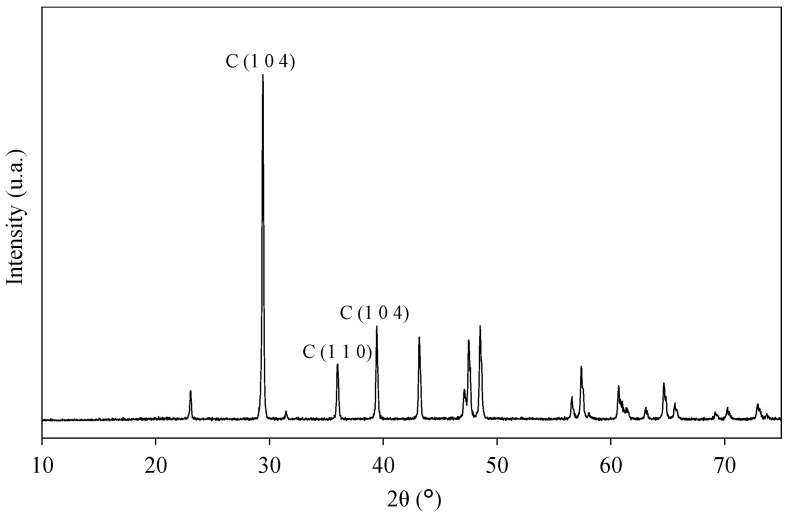
XRD analysis of recovered calcium carbonate.

Moreover, in order to complete the characterization, XRD analysis was carried out (Figure 10).

The result of this XRD analysis was compared with the reference code 98.002.0179 of the ICDD database, where all signals corresponded to calcite with no residual signal, confirming the high purity of the recovered calcium carbonate. In addition, it was compared with other diffractograms in the literature presenting the same signals and appearance [41], demonstrating that CaCO_3_ was recovered completely pure and could be reused as a PU filler.

### 3.6. Synthesis and Characterization of the Synthesized Foams Using Recovered Fillers

The recovered styrene–acrylonitrile (SAN) and recovered calcium carbonate (CaCO_3_) obtained were then reused in the synthesis of new PU foams without altering the rest of the recipe. The effect of this SAN and CaCO_3_ replacement on the foaming process and foam quality was evaluated. The blow-off time during the foaming process was logged; also, the density, the air resistance, the CLD hardness 40%, the compression set 50% and the ball rebound (elasticity) were measured.

Table 7 shows the results of the characterization of a PU foam employing the SAN polymer polyol and of another employing the recovered SAN and raw polyol as replacements of this SAN polymer polyol.

The replacement of the SAN polymer polyol (a dispersion of 44% SAN solids in 56% of the conventional polyol) by the recovered SAN together with the conventional polyol seems not to have a considerable effect on the foaming process and the resulting density.

With the recovered SAN, the foam is much more closed, resulting in a crimp of foam as indicated by the high air resistance values. This may be improved as demonstrated in Section 3.3 by introducing isocyanate mixtures, leading to open cell structure foams with a lower air resistance value. The foam becomes slightly softer, while the compression set 50% and elasticity become slightly worse. This is probably due to the fact that the recovered SAN was not as well dispersed in the polyol as the original SAN.

On the other hand, PU foams were synthesized employing commercial or recovered CaCO_3_, without altering the rest of the recipe. Table 8 shows the recipe and characterization of these foams.

The replacement of the standard using CaCO_3_ with the recovered CaCO_3_ seems not to have a considerable effect on the foaming process, density and foam properties. With the recovered CaCO_3_, the foam is much more closed, resulting in a crimp of foam as indicated by the high air resistance values, and the elasticity becomes slightly worse. The use of isocyanate mixtures could help to obtain lower air resistance values, as demonstrated in Section 3.3.

Thus, it has been demonstrated that the recovered SAN and CaCO_3_ can be used as filler material for the synthesis of new polyurethane foams, replacing commercial fillers, or could even be used in other applications.

## 4. Conclusions

The main conclusions of this research are listed below.

- The use of DABCO as a catalyst and DEG as the glycolysis agent allowed us to achieve the glycolysis of flexible polyurethane foam waste composites in the split phase, recovering the styrene–acrylonitrile and calcium carbonate contained as fillers.
- The use of DABCO as a catalyst allowed us to reduce the catalyst concentration and the mass ratio of polyurethane to glycol while maintaining the split phase to 0.1% and 1:1, respectively.
- The recovered polyol presented higher purity than those reported in previous works, after which the purification of the upper phase was obtained with an approximate purity of 99% by GPC and a hydroxyl value of 48 mg KOH/g.
- The applicability of the raffinate recovered polyol for the synthesis of polyurethane foams was demonstrated, but percentages from 50% by weight led to foams with a closed-cell structure and therefore with higher air resistance values.
- The closed-cell structure was corrected by using a blend of TDI65 and TDI80 isocyanates, finding an optimized ratio of 70 and 30%, respectively.
- Foams with up to 70% recovered polyol with all properties within the specification were synthesized using the optimized isocyanate blend.
- Styrene–acrylonitrile and calcium carbonate, recovered from foam waste, were proved to be suitable for the synthesis of new polyurethane foams, with properties quite similar to the reference one for the case of CaCO_3_. However, in both cases, the foams presented a closed-cell structure, which could be corrected by the addition of TDI65 to TDI80.

## Figures and Tables

**Table 1:** Glycolysis reaction conditions and recipe.

Reaction Conditions		Reaction Recipe	
Ratio PU: glycolysis agent	1: 1	Polyurethane foam	450 g
Catalyst concentration	0.1 wt%	data	
Reaction temperature	200 °C	Catalyst (DABCO)	0.45 g
Stirrer type	Rushton		
Stirring speed	300 rpm	Diethylene glycol	450 g
Feeding time	1 h		
Reaction time	3 h		

**Table 2:** Recipe of the synthesized foams.

Compound	p/p w
Polyol	100
Water	4.22
Surfactant	1.2
Amine catalyst	0.22
Metal catalyst	0.32
Isocyanate TDI80	53.77
Index	110

**Table 3:** Concentration in wt% of the different compounds in the UP, IP and BP at the end time of the reaction.

		Concentration (wt%)			Molecular Weight (g/mol)
		Upper Phase	Intermediate Phase	Bottom Phase	
SAN	Peak I	0	21	2.5	70,000
Polyol	Peak II	89.6	39	5.2	3500
Reaction by-products	Peak III	5.9	25	57.1	700
	Peak IV				475
	Peak V				320
DEG	Peak VI	4.5	15	35.2	106

**Table 4:** Composition, hydroxyl number, water content and viscosity of recovered polyol.

Composition (wt%)	SAN	0
	Polyol	99.1
	Reaction by-products	0.9
	DEG	0
Hydroxyl number (mg KOH/g)		48.4 ± 0.5
Water content (wt%)		0.07
Viscosity (Pa·s)		1.5

**Table 5:** Air resistance values of each foam synthesized with 100% of recovered polyol and different isocyanate mixtures.

TDI80 (%)	TDI65 (%)	Air Resistance (cm H_2_O)
100	0	85
90	10	84.5
50	50	85.5
30	70	10
0	100	6.8

**Table 6:** Characterization of the different foams synthesized using different proportions of virgin and recovered polyol.

Recovered Polyol (%)	0 *	50	60	70	80	90	100
Blow-off time (s)	86	87	86	84	85	85	82
Density (kg/m^3^)	24.7	25.4	25.1	25.5	25.8	26.4	24.5
Air resistance (cm H_2_O) min	6.2	4.9	5.1	5.0	8.1	6.8	9.4
Air resistance (cm H_2_O) max	6.7	5.2	5.5	6.7	8.5	7.0	10.0
CLD hardness 25% (kPa)	3.4	3.5	3.8	4.1	4.2	4.4	4.1
CLD hardness 40% (kPa)	3.9	4.2	4.3	4.8	4.9	5.0	4.7
CLD hardness 60% (kPa)	5.9	6.8	7.0	7.9	8.0	8.3	7.8
Ball rebound (%)	43	37	37	36	33	34	34
Compression set 50% (%)	2.1						1.9
Compression set 75% (%)	4.1						4.6
WCS 50 °C 95% RH (%)	7.0						9.6
Tensile strength (kPa)	88						73
Elongation at break (%)	139						91
Tear resistance (N/cm)	3.9						3.6

* Foam with 0% of recovered polyol was synthesized just with TDI80.

**Table 7:** Characterization of PU foams employing SAN polymer polyol and recovered SAN.

Conventional polyol	75	89
Conventional SAN polymer polyol	25	-
Recovered SAN	0	11
Isocyanate TDI80	100%	100%
Blow-off time (s)	82	85
Density (kg/m^3^)	24.9	24.6
Air resistance (cm H_2_O) min	26.1	85.5
Air resistance (cm H_2_O) max	49.7	85.8
CLD hardness 25% (kPa)	5	3.8
CLD hardness 40% (kPa)	5.8	4.5
CLD hardness 60% (kPa)	9.1	7.3
Compression set 50% (%)	3	3.4
Ball rebound (%)	34	20

**Table 8:** Characterization of PU foams employing commercial and recovered CaCO_3_.

Conventional polyol	86	86
Commercial CaCO_3_	14	0
Recovered CaCO_3_	0	14
Isocyanate TDI80	100%	100%
Blow-off time (s)	80	88
Density (kg/m^3^)	24.2	24.3
Air resistance (cm H_2_O) min	33	85.4
Air resistance (cm H_2_O) max	40.5	85.6
CLD hardness 25% (kPa)	4.3	4.3
CLD hardness 40% (kPa)	4.9	4.9
CLD hardness 60% (kPa)	7.9	7.8
Compression set 50% (%)	3.2	3.2
Ball rebound (%)	34	19

## Data Availability

The original contributions presented in the study are included in the article, further inquiries can be directed to the corresponding author.
